# The influence of graphic warning labels on efficacy beliefs and risk perceptions: a qualitative study with low-income, urban smokers

**DOI:** 10.1186/s12971-016-0088-5

**Published:** 2016-07-27

**Authors:** Erin L. Mead, Joanna E. Cohen, Caitlin E. Kennedy, Joseph Gallo, Carl A. Latkin

**Affiliations:** 1 Department of Health, Behavior and Society, Johns Hopkins University, Bloomberg School of Public Health, 624 N. Broadway, Baltimore, MD 21205 U.S.A.; 2 Department of Health, Behavior and Society, Institute for Global Tobacco Control, Johns Hopkins University, Bloomberg School of Public Health, 2213 McElderry St., Fourth Floor, Baltimore, MD 21205 U.S.A.; 3 Department of International Health, Johns Hopkins University, Bloomberg School of Public Health, 615 N. Wolfe St., Baltimore, MD 21205 U.S.A.; 4 Department of Mental Health, Johns Hopkins University, Bloomberg School of Public Health, 615 N. Wolfe St., Baltimore, MD 21205 U.S.A.; 5 Department of Behavioral and Community Health, University of Maryland School of Public Health, Tobacco Center of Regulatory Science, SPH Building (255), 4200 Valley Drive, College Park, MD 20742-2611 U.S.A.

## Abstract

**Background:**

Health communication theories indicate that messages depicting efficacy and threat might promote behavior change by enhancing individuals’ efficacy beliefs and risk perceptions, but this has received little attention in graphic warning label research. We explored low socioeconomic status (SES) smokers’ perceptions of theory-based graphic warning labels to inform the development of labels to promote smoking cessation.

**Methods:**

Twelve graphic warning labels were developed with self-efficacy and response efficacy messages paired with messages portraying high, low, or no threat from smoking. Self-efficacy messages were designed to promote confidence in ability to quit, while response efficacy messages were designed to promote confidence in the ability of the Quitline to aid cessation. From January – February 2014, we conducted in-depth interviews with 25 low SES adult men and women smokers in Baltimore, Maryland, U.S. Participants discussed the labels’ role in their self-efficacy beliefs, response efficacy beliefs about the Quitline, and risk perceptions (including perceived severity of and susceptibility to disease). Data were analyzed through framework analysis, a type of thematic analysis.

**Results:**

Efficacy messages in which participants vicariously experienced the characters’ quit successes were reported as most influential to self-efficacy beliefs. Labels portraying a high threat were reported as most influential to participants’ perceived severity of and susceptibility to smoking risks. Self-efficacy messages alone and paired with high threat were seen as most influential on self-efficacy beliefs. Labels portraying the threat from smoking were most motivational for calling the Quitline, followed by labels showing healthy role models who had successfully quit using the Quitline.

**Conclusions:**

Role model-based efficacy messages might enhance the effectiveness of labels by making smokers’ self-efficacy beliefs about quitting most salient and enhancing the perceived efficacy of the Quitline. Threatening messages play an important role in enhancing risk perceptions, but findings suggest that efficacy messages are also important in the impact of labels on beliefs and motivation. Our findings could aid in the development of labels to address smoking disparities among low SES populations in the U.S.

## Background

The implementation of pictorial labels warning about the health consequences of smoking on cigarette packaging, called graphic warning labels, is an important element of global tobacco control policy [1, 2]. Graphic warning labels are more effective than text-only labels at promoting smoking cessation behaviors, including increased calls to a national Quitline and quit attempts [3–5]. They can be a prominent source of health information to promote changes in attitudes, beliefs, knowledge, intentions to quit, and behaviors such as quit attempts [4, 6–10].

Despite the growing evidence of the superior effectiveness of graphic to text-only warning labels, limited research has examined the content of labels that is most persuasive for smokers, other than formatting characteristics such as real photographs versus cartoons [4, 11–15]. Much of the development of label content has relied on fear appeals using vivid depictions of the negative consequences of smoking [4]. Research has shown that fear and other strong negative affective responses (such as worry) to labels are associated with cognitive reactions (e.g., believability), greater risk perceptions, lower desire to smoke, positive feelings towards quitting, intentions to quit, and future self-reported quitting behaviors [9, 16, 17]. Strong emotional reactions are associated with improved memory of labels and increased neurological responses to labels [18, 19]. However, there is considerable variability in how smokers and nonsmokers rate the effectiveness of highly vivid labels [20]. Other researchers have raised concerns that these highly threatening messages may cause a defensive reaction that will continue or increase smoking, particularly among those with low confidence to take protective action [21, 22]. There is need to further study the content of warning labels to determine what elements are most persuasive for cessation and do not lead to defensive reactions.

The application of the extended parallel process model (EPPM) to labels can be highly useful to understand the apparent contradictory findings of highly vivid labels’ effects on smoking behaviors, and to aid in the development of persuasive labels. According to EPPM, labels would be most effective when portraying both a threat that arouses fear as well as the efficacy of a recommended action to mitigate the threat [23, 24]. The threat message—characterized by severity of and susceptibility to a health condition—motivates action through fear. However, the efficacy message—characterized by the effectiveness of the recommended action to reduce risk (response efficacy) and the individual’s ability to perform the action (self-efficacy)—determines whether the individual will engage in fear control behaviors (defined as coping behaviors to reduce fear such as avoidance) or danger control behaviors (defined as adoption of the recommended action). A meta-analysis found an interactive effect between threat and efficacy, such that threat was associated with positive behavior change only when efficacy was high, and vice versa [21]. Indeed, threat with low efficacy may be associated with negative behavioral outcomes, suggesting the importance of efficacy information on labels [21, 25, 26]. A more recent study did not find evidence of this “boomerang” effect of graphic warning labels on quit attempts among smokers with low self-efficacy [27], but efficacy beliefs appear to have an important moderating effect on reactions to labels and subsequent quit attempts [27, 28].

These studies do not shed light on how labels could be used to increase efficacy beliefs, and thereby enhance the persuasiveness of the message. Few studies have examined the effect of labels on self-efficacy and response efficacy beliefs, and we could find none that used evidence-based theory to develop and explore perceptions of efficacy messages on graphic warning labels. The limited evidence has shown very little impact of existing labels on increasing smokers’ self-efficacy beliefs [17, 26, 29–31]. This might be due to the lack of development of efficacy messages [32]. An experimental study found that graphic warning labels had no effect on changing self-efficacy beliefs compared to text-only labels [26]; however, the text (“Studies have shown that tobacco can be harder to quit than heroin or cocaine”) might be more likely to decrease self-efficacy than increase it. All warning labels include a Quitline telephone number for cessation help, but limited research has examined the influence of labels on perceived effectiveness for quitting. Waters et al. [33] found that U.S. adolescent and adult nonsmokers and smokers had low confidence in the efficacy of the Quitline when viewing it on a graphic warning label. Response efficacy messages designed for labels might increase confidence in the Quitline. In sum, our knowledge is limited about the types of messages on graphic warning labels that can promote self-efficacy and response efficacy beliefs to encourage cessation.

Following Bandura’s work [34], we identified three types of self-efficacy messages for this study: (1) *mastery experiences*; (2) *vicarious experiences*; and (3) *social persuasion.* In **mastery experiences**, individuals build their self-efficacy by performing a task successfully. These tasks must be attainable and can be small steps on the road to a significant behavior change, but they require perseverance to overcome obstacles in order to build resilient self-efficacy. Although the labels cannot provide that experience directly to smokers, mastery experience labels were designed to suggest small, attainable behaviors in which smokers could engage to help build efficacy, such as delaying smoking. The **vicarious experience** (or social modeling) approach occurs when individuals see others who are similar to themselves succeed in completing a task through perseverant effort and are rewarded for that effort. Observing modeling increases individuals’ expectations that they can also perform the behavior and will have a positive outcome. Vicarious experience labels were designed to tell the story of a character, such as how a character quit smoking. In **social persuasion**, individuals are persuaded that they have the skills and capability to perform the behavior through greater, perseverant effort, even in the face of obstacles. Social persuasion labels encouraged the person to exert greater effort towards the goal, such as affirming their power to quit.

Investigating threat and efficacy messages on graphic warning labels might be particularly important among low socioeconomic status (SES) populations given the evidence that these groups are less able to access health information than high SES groups [35–37]. Cultural and literacy factors influence how information is accessed, processed, and used by groups [35, 38]. Graphic warning labels would provide ubiquitous access to health information for smokers who carry cigarette packs, regardless of SES, but the effectiveness of specific content might differ by socioeconomic indicators [11, 39]. Moreover, low SES smokers may perceive that they do not have the resources to quit or would be unable to quit in the face of cognitive barriers such as stress. Labels could provide information to enhance their efficacy to quit and overcome these challenges. This is imperative, given that low SES individuals are more likely to be current smokers and less likely to quit or make a quit attempt [40–43]. To address health equity gaps, further research is needed on low SES smokers’ perceptions of graphic warning labels and their threat and efficacy content.

Our previous qualitative research with this population of low-income, urban smokers found that labels identified as highly motivational influenced risk perceptions, generated affective reactions such as fear and concern, had hopeful messages, or used role models for quitting [44]. This study extends our previous work by looking more specifically at the pathway by which the labels might influence motivation to quit through risk perceptions and efficacy beliefs, guided by EPPM. We look at the threat and efficacy messages of the labels to explore their role in risk perceptions and efficacy beliefs. We developed efficacy messages based on health communication and behavioral theories and explored perceptions of labels with a threat message, an efficacy message, and a threat + efficacy message. Our aim was to examine what types of threat and/or efficacy messages might be the most promising for changing risk perceptions and efficacy beliefs in this high priority population of smokers.

## Methods

### Sample

Participants were recruited from a population of current smokers who completed a survey for the Tobacco Influences in the Drug Environment (TIDE) study, which aimed to identify communication channels that promote tobacco use and cessation among low-income smokers in Baltimore, Maryland. TIDE recruitment took place in low-income neighborhoods through street outreach and word-of-mouth by trained staff from the Lighthouse Studies at Peer Point. This population was chosen because of its high smoking prevalence. In Baltimore, smoking prevalence is highest among those with the lowest income (35 %, <$15,000 annual income) and education (34 %, less than college degree) [45], and smoking prevalence is as high as 58 % in some groups [46]. TIDE inclusion criteria were being 18 years or older, having smoked ≥100 cigarettes in lifetime, and having smoked cigarettes in the past 30 days.

All TIDE participants were eligible for our qualitative study. Participants were chosen using purposive sampling by gender and age group (18–39 and ≥40 years) to capture variations among younger and older smokers as well as among men and women who might have different health concerns. All TIDE participants approached for the qualitative study agreed to participate.

### Data collection

The first author (E.L.M.) conducted in-depth interviews in a private office at the Lighthouse from January to February 2014. The interviews lasted 1–2 h and were audio recorded. Participants were shown 12 graphic warning labels in random order and asked about their cognitive and affective reactions to each label (see Appendix for interview guide). They were then asked to select which labels showed the highest level of harm from smoking (perceived severity), showed a health effect likely to happen to them if they did not quit or made them worry the most about their smoking (perceived susceptibility), made them feel confident that they could quit if they wanted to (self-efficacy belief), and would motivate them to call the Quitline (response efficacy belief). Data on age, gender, race, marital status, education, employment status, income, smoking frequency, and quitting behavior were also collected.

### Ethics approval

Participants provided written informed consent prior to the interview and were compensated $25. The Johns Hopkins University Bloomberg School of Public Health Institutional Review Board approved the study.

### Label development

For the study, graphic warning labels were either adapted (with permission) from existing labels (from Canada, the U.S., Brazil, and Australia) or created (Table 1). Labels were standardized to include a warning statement at the top, a picture on the left, and the U.S. Quitline number. The warning statements mandated by U.S. law were used whenever possible. In addition, some labels contained text describing either the threat from smoking or an efficacy message, which are described below.

**Table 1:** Characteristics of the threat level and efficacy messages on graphic warning labels

Label #	Label image	Threat level	Efficacy message	Health message
1		Low	Self-efficacy: Vicarious experience	Negative health effects of smoking for smokers
2		High	Self-efficacy: Mastery experience	Negative health effects of smoking for smokers
3		Low	Weak	Negative health effects of smoking for smokers
4		High	Response efficacy: Quitline’s helpfulness for quitting	Negative health effects of smoking for smokers
5		High	Weak	Negative health effects of smoking for others
6		Low	Self-efficacy: Mastery experience	Negative health effects of smoking for others
7		Low	Response efficacy: Quitting to improve health	Negative health effects of smoking for others
8		High	Self-efficacy: Social persuasion	Negative health effects of smoking for others
9		None	Response efficacy: Quitting to improve health	Benefits of quitting for smokers
10		None	Self-efficacy: Vicarious experience	Benefits of quitting for smokers
11		None	Self-efficacy: Social persuasion	Benefits of quitting for others
12		None	Response efficacy: Quitline’s helpfulness for quitting	Benefits of quitting for others

Following EPPM [23], labels were developed with different threat and efficacy messages. The threat message varied on the level of threat portrayed, i.e., high, low, and none. Based on categorization used previously [47], labels with a vivid picture of the negative effects of smoking were categorized as portraying a high threat level, whereas labels with a nonvivid picture of the negative effects were categorized as low threat. Labels with a picture relating to a positive message about quitting were categorized as no threat. In addition, the threat message varied by the type of health effect (e.g., cancer, secondhand smoke effects). Three types of self-efficacy messages were designed (as described above): mastery experiences, vicarious experiences, and social persuasion. Response efficacy messages addressed the effectiveness of quitting on improving health and calling the Quitline in aiding cessation. Efficacy messages were combined with different threat levels to explore how labels with varying combinations of threat and efficacy influence efficacy beliefs. Labels varied on their portrayal of health effects of smoking to smokers and others and of benefits of quitting for smokers and others [44].

An initial set of eight labels was developed using the methods and classifications described above. A meeting was held to discuss the labels with staff of the Lighthouse Studies at Peer Point, which is a community-based research center that works with low SES populations with a high burden of injection drug use and HIV [48]. The Lighthouse staff have a significant amount of expertise and experience working with this population, and so their input on the labels was highly valuable as a first step for label development. During the meeting, the staff discussed and compared the labels on the ability to grab attention, how understandable the message was, the layout and design (including the level of threat portrayed), the possible behavioral effects of the labels on the population, and suggested improvements. Using the feedback, 12 labels were revised or developed. They were pilot tested with three participants through in-depth interviews. Based on the results, further revisions were made to pictures and text to enhance clarity and threat and efficacy messages; revised labels were piloted with another two participants. The 12 labels were then finalized.

### Data analysis

In *Atlas.ti* v7, the transcribed interviews were coded by the first author (E.L.M.) with a coding scheme that was developed using a combined deductive and inductive approach with input from two co-authors (J.E.C. and C.E.K.). Analytic memoing was conducted to reflect on emerging themes or issues, including deviant cases. Deviant cases were exceptions to the narrative that were noted and described to test the emerging findings. The framework method was used for analysis of the coded transcripts, which is a thematic analysis using a matrix structure to systematically reduce the data [49]. Following this method, codes were grouped into broader categories to begin the process of data abstraction, such as a category for efficacy-related codes. Next, data were charted into the framework matrix to provide accurate summaries by participant, category, and label. For example, responses were summarized for the codes within the response efficacy category for each participant and label. Broader themes were developed by comparing codes and categories within and across cases with special attention to deviant cases. To enhance rigor and transparency [49], the data were summarized by case within the matrix, thus keeping the data within the rich context of each case. The matrix structure facilitated the identification of patterns and included references to specific transcript lines to easily ascertain the evidence supporting the themes.

## Results

### Study sample

Characteristics of the study population have been reported elsewhere [44]. In brief, the 25 participants were on average 45 years old, 22 were African American, and 13 were women. Most had completed high school/GED (*n* = 11) or less (*n* = 12), earned an income < $10,000 during the previous year (n = 16), were retired or unable to work for health reasons (*n* = 16), and were either single (*n* = 12) or married/partnered (*n* = 12). Twenty-three reported smoking every day in the previous 30 days, and 11 reported smoking <1 pack per day. Over half had ever tried to quit, 11 had made ≥1 quit attempts in the previous 12 months, and eight were currently trying to quit.

### Reactions to efficacy messages

Participants were asked about their reactions to social persuasion, mastery experience, and vicarious experience self-efficacy messages on the labels (Table 1). Many participants responded favorably to social persuasion messages, designed to persuade individuals that they had the ability to quit. Participants stated these messages were credible and helpful: “[If] you want to be around your kids and stuff, you need to quit. It gives you a lot of hope… I feel good just seeing you’ve got the power to quit, like you can do it. Like saying they did it and they’re happy now” (man, 31 years old). For several participants, the message reminded them of their ability to quit, thus influencing their self-efficacy beliefs. Several other participants reported that these messages were not helpful or credible, citing difficulties in overcoming nicotine addiction.

Most participants reacted negatively to mastery experience messages, designed to suggest small behavioral steps towards cessation that individuals could master to increase their self-efficacy. One main reason was their lack of credibility – they reported that the behavioral step would not be effective for cessation. In addition, participants did not think they had the ability to master the behavioral step and would need additional help; in other words, they had low self-efficacy. For example, when asked about a message suggesting the delay of the day’s first cigarette to facilitate cessation, one woman (46 years old) stated: “No. I don’t think so. I ain’t got that happening… when I get up in the morning I have a cigarette. Then in like a good, I ain’t even going to say a half an hour, probably 15–20 min I go smoke another one.” Several participants stated that the mastery experiences were helpful and they might try the behavioral steps.

Many participants reacted positively to vicarious experience messages, which were designed to tell the stories of characters who quit. Participants reported that the characters’ quit methods would be helpful for quitting, and often added methods, such as removing ashtrays, enforcing smoke-free home policies, and using nicotine replacement therapy. Moreover, they described the characters as role models to admire and emulate: “That one about Michael quitting before he set his quit date, he looks at his quit date and get rid of all his cigarettes. I think I can do that. When I really, really feel like it need to be done which is now, I think I can do it” (man, 47 years old).

However, several participants stated that vicarious experience messages were not helpful because the quit method was ineffective. Underlying this statement for several participants seemed to be a belief that they did not have the ability to use that method successfully, that is, low self-efficacy: “No. I don't think that's true… I generally had to have something to help remove those urges, to stop those urges from being strong… yeah, you ain't just going to just stay busy and stop smoking. That's not going to happen” (woman, 55 years old). This participant not only questioned the validity of the quit method but also her ability to use it without succumbing to her urges. The characters failed to be adequate role models of cessation for several participants.

Reactions to the response efficacy messages about quitting were mixed. One label discussed the efficacy of quitting for improving children’s health. All but one participant stated that the message was true. Although they stated the message was helpful for smokers, many participants discussed the need to stop smoking in front of children, rather than the need to quit all together. The other label discussed the efficacy of quitting on reducing the smoker’s risk of heart attacks and cancer. Most participants stated that they believed the message, particularly about cancer risk. However, doubts were raised about the credibility of reducing their risk of heart attacks and how long it takes for their risk to decrease. Several participants doubted that quitting would improve their health. They discussed other risk factors that they believed to be as or more important than smoking, including diet, physical activity, and being “just prone to these diseases.”

Most participants knew little about the Quitline, such as the services it provides, the cost of the services, and if it is automated. A few participants had previous experience with the Quitline or knew someone who had used it. Two labels discussed the Quitline aiding cessation. Positive and negative reactions about the credibility and helpfulness of the messages were almost evenly split. Reasons for positive reactions included that the Quitline could provide necessary help and support from former smokers, access to free services nearby, and the knowledge that others were able to quit using the Quitline. Participants were not always certain that the Quitline fulfilled these criteria, but reported that it would be helpful if it did. Reasons for negative reactions included that the services provided would not be sufficient for quitting, a desire for face-to-face communication (rather than via telephone), and the Quitline would not help unless the person had a strong desire to quit.

### Role in self-efficacy beliefs

Participants were asked which labels made them feel more confident that they could quit smoking if they wanted to (i.e., quitting self-efficacy). No clear pattern emerged by the type of self-efficacy message, but labels with a positive message about the benefits of quitting for others and for self were reported as the most influential for efficacy beliefs (Table 2). Participants’ discussions showed that they vicariously experienced the situations portrayed by the characters and role modeling played a role in shaping their self-efficacy beliefs. Five of the labels showed one or more characters who had quit smoking (labels #1, 9–12), and the majority of participants discussed at least one of these characters as a role model for quitting and living a healthy lifestyle: “This one [character on label #10] makes me proud that they was able to do it and I can do it too” (woman, 58 years). The two labels selected most often were both positive messages about quitting, but one had a social persuasion message (label #11) and the other had a response efficacy message about quitting (label #9). The major reasons for selecting the social persuasion label were that the characters were role models showing the benefits of quitting and the social persuasion message was motivating: “They triumphed. They’ve proven that people can stop smoking and that whole families can do it… Cigarette smoking can be stopped. It’s only an urge; that’s all it is” (man, 51 years old). The overwhelming reason for selecting the response efficacy label (#9) was that the character was a positive role model for quitting and looked healthy after quitting: “You know, it make you say, ‘Wow, if he could quit at such a young age, you know, I should be able to quit’” (woman, 39 years old). These statements illustrate how vicariously experiencing a quit attempt can influence self-efficacy.

**Table 2:** Frequency of selection of graphic warning labels for risk perceptions and efficacy beliefs (*n* = 25)

	Quitting self-efficacy	Quitline response efficacy	Perceived severity	Perceived susceptibility
Label 1	5	6	11	16
Label 2	7	9	20	17
Label 3	6	9	17	16
Label 4	5	7	17	18
Label 5	3	4	22	11
Label 6	2	3	4	7
Label 7	5	5	10	5
Label 8	4	5	12	15
Label 9	8	3	0	2
Label 10	6	4	0	3
Label 11	9	4	1	3
Label 12	6	8	1	3

Six participants reported that none of the labels increased their quitting self-efficacy. Four of them had never tried to quit; in contrast, 12 of the 19 participants who selected ≥1 labels had tried to quit. Several had no desire to quit and were resistant to the messages, doubting their credibility. Others expressed their desire to quit at some point in the future but had low self-efficacy to quit at the moment:

Cravings can stop you from quitting… because if you got these strong cravings and you know that you really want it, you ain't going to stop. You ain't going to stop… You got your mind set on quitting but then here comes something else that make you [say], “oh, I need a cigarette” (man, 39 years old).

One participant reported that none of the labels affected her self-efficacy because she already had high self-efficacy.

We also explored how participants’ perceptions might have been influenced by the portrayal of a high threat (i.e., containing vivid pictures), low threat or no threat from smoking-related conditions. When a label contained a self-efficacy message, participants most often reported that labels with no threat, followed by high threat, influenced their self-efficacy beliefs. Participants reported that high threat labels showed the negative effects from smoking and motivated them to quit to avoid those conditions and improve their overall health. In contrast, participants stated that no threat labels showed characters who were role models for quitting and showed the effectiveness of quitting on improving health. They also stated that the labels made them more confident to overcome obstacles to quitting (e.g., cravings) and the self-efficacy text was motivating.

### Role in response efficacy beliefs about the quitline

Participants were asked which labels would motivate them to call the Quitline (Table 2). They most often reported being motivated by labels that portrayed the threat of smoking to themselves, including the condition looked severe or “scary”, concern and fear about their own vulnerability to that disease, a desire to avoid the disease, and the credibility of the message.

In addition to threatening labels, one of the labels most frequently identified as motivational for calling the Quitline was the label depicting a happy couple with a response efficacy message about the Quitline and a message about the benefits of quitting for others (label #12). Participants described how the label showed that the Quitline works and gives smokers their best chance to quit. They described how the couple in the picture used the Quitline to quit smoking, and wanted to be happy and healthy like the characters: “Yeah, the Quitline helped make them stronger, and they were able to move on, possibly help others” (man, 44 years old).

Seven participants reported that none of the labels motivated them to call the Quitline. They described how the Quitline would not work for them, they have no need for it, or they did not believe that it has helped others. One participant stated that she does not need it and has sufficient strength to quit on her own. Another woman previously used the Quitline to quit and is planning to use it again; therefore, the labels provided no additional motivation.

### Role in risk perceptions

Participants were asked which labels influenced their perceived severity of and susceptibility to smoking-related conditions (Table 2). They most often reported that the labels portraying a high threat influenced their perceived severity of and susceptibility to smoking, followed by low threat labels. Labels depicting a threat to self were more influential than labels depicting a threat to others. All participants perceived high severity from at least one high threat label. The picture was most often the reason for the label’s influence, and other major reasons included negative emotional reactions (e.g., scared, anxious) and the clear provision of information. Some participants reported these labels as influential because they contained new information and because of the potential long-term health outcomes (i.e., diminished quality of life, irreparable physical damage, and death).

Participants reported that high and low threat labels influenced their perceived susceptibility because they were concerned about these conditions and wanted to prevent them, or they perceived high severity of the conditions. Other major reasons stated were that they have, or know someone who has, a similar condition, and the labels stimulated them to contemplate how much physical damage smoking had caused to their bodies: “You wonder how close you is to being like these people. You might don’t even know it” (man, 47 years old). Several participants also selected labels showing conditions that they stated were inevitable if they continued to smoke.

In a notable case, a woman (39 years old) qualified her selections by stating that smoking cigarettes is not the main cause of these conditions. She was the only participant to say that none of the labels made her feel susceptible and to doubt that smoking harms children. Indeed, she often criticized the labels’ credibility and expressed frustration and anger at the perceived misinformation: “It’s all different kind of lung diseases out there and it don’t come from tobacco. So I don’t know where they getting this crap from, but I think they need to redo their research all over again.” She and some other participants described the inevitability of disease even if they quit because of other exposures, such as environmental toxins and secondhand smoke.

## Discussion

Our study explored perceptions of graphic warning labels among adult low-income smokers in Baltimore, MD. Using health communication and behavioral theories [24, 34], we developed several types of efficacy and threat messages and explored the perceived influence of the labels on risk perceptions (perceived severity and susceptibility), self-efficacy beliefs (confidence to quit), and response efficacy beliefs (effectiveness of the Quitline). Our study is novel in its use of behavioral and communication sciences to develop and study graphic warning labels on tobacco packs for a high priority population of smokers.

We found that, when asked which labels influenced their self-efficacy beliefs, low income smokers selected most often labels with efficacy messages and a non-threatening image (i.e., showing the benefits of quitting for self and others), followed by a high-threatening image. Reasons included enhanced feelings of confidence from self-efficacy messages, vivid pictures, and desires to avoid disease and be healthy. This finding illustrates the complex interplay between risk perceptions and efficacy beliefs in the influence of graphic warning labels. Research has shown that, in general, higher threat messages are more persuasive and accepted than lower threat messages only among individuals with high self-efficacy [50, 51]. One study found that labels increased intentions to quit only among smokers with stronger quitting self-efficacy beliefs [26]. Our research is the first to show that theory-driven self-efficacy messages on graphic warning labels, even in the absence of threat, can play a role in low income smokers’ confidence to quit.

Public health practitioners have recommended the development of labels with efficacy messages [32, 52]. While previous studies have found limited impact of labels on efficacy beliefs [4, 26, 29, 30], their findings might be due to the lack of development of theory-driven self-efficacy messages. To fill this gap, we developed three types of self-efficacy messages (i.e., social persuasion, mastery experience, and vicarious experience) and two types of response efficacy messages (i.e., about quitting and the Quitline) and explored reactions to these messages and their role in efficacy beliefs. We found that participants’ self-efficacy beliefs seemed to be most influenced by vicariously experiencing the quit successes and benefits of quitting of the characters pictured on the labels. Mastery experiences appeared to have little or no effect, which might be due to the difficulty of conveying these experiences on a label. According to Bandura, mastery experiences involve actually enacting a behavior and experiencing a successful outcome, not merely observing the behavior of others, which is challenging to portray on a label.

Observing others perform actions and the consequences of those actions is one way that individuals learn, and observing the behaviors of role models in the media can not only teach new skills but also enhance self-efficacy to perform those behaviors [53]. For example, the use of role models has been shown to increase self-efficacy and intentions to perform breast self-examinations [54], rehabilitation self-efficacy and outcomes following knee surgery [55], and smoking cessation during pregnancy [56]. Our study is the first in the published literature to suggest that low income smokers might vicariously experience the quit successes and benefits of quitting among characters on graphic warning labels, which might lead to increasing self-efficacy.

Narrative communication, which describes events and characters to promote a particular message, is an effective means to engage the audience in vicariously experiencing characters’ behaviors and outcomes, thus overcoming message resistance and promoting acceptance [38]. However, studies on the use of testimonials on graphic warning labels, which are narratives of real smokers’ experiences with smoking-related conditions, have shown mixed results regarding their perceived effectiveness [4, 15, 20, 47, 57]. Testimonial labels might be most effective among smokers with greater self-efficacy [29] and low educational attainment [39]. Overall, research on testimonial labels is limited because it only examined individuals suffering from smoking-related conditions, rather than their quit successes. Our study shows that low income smokers also vicariously experience characters’ quit successes, which might be an important pathway for labels to enhance quitting self-efficacy. However, the characters were not adequate role models for all participants. Research is needed to develop and test graphic warning labels with appropriate and realistic role models for cessation success using a narrative format.

Our findings also show the challenges to improving perceptions of the Quitline’s utility for cessation. In 2007 about half of the U.S. population was aware of the Quitline but only 9 % of aware smokers had called, suggesting that factors other than awareness are important influences [58]. Increasing knowledge of the Quitline’s services in this population of low-income, urban smokers might be one step towards improved response efficacy beliefs about the Quitline, but is not sufficient motivation to call. Our text-based response efficacy messages had limited success in influencing efficacy beliefs about the Quitline. The response efficacy label that had the most success informed the audience that many others had used the Quitline and showed a picture of a healthy, happy couple who had used it. These messages appealed to our study population at least in part due to the role modeling exhibited by the characters, aspiration to be healthy and happy like the characters, and knowledge that it has worked for many others (i.e., perceived norms). Similar to other research [59], our participants expressed beliefs that they had no need for assistance, the Quitline would not be effective, and the telephone modality would not work. Overall, the cognitive factors that predict Quitline calls are unclear [60]. Evidence suggests that smokers prioritize lay knowledge of the effectiveness of assisted vs. unassisted cessation over expert medical knowledge and ascribe value and self-worth to quitting without assistance [61]. Messages should be developed for labels that tap into these cultural and personal values. For example, testimonials from former smokers, their initial misgivings about the Quitline, and how it helped them could be useful package inserts. Combined with formatting to increase the salience of Quitline number and message [62], this approach might be the most effective strategy to improve perceived efficacy and use of the cessation service.

In addition, our study explains a pathway through which labels might influence cessation among low-income smokers—by enhancing perceived severity of and susceptibility to smoking-related conditions. When asked which labels affected their risk perceptions the most, participants selected high threat labels, followed by low threat labels, because of the vivid picture, negative affective reactions, and information provided. These findings are consistent with other evidence that suggests vivid depictions of the physical effects from smoking are most effective in changing smoking-related attitudes and behaviors [4, 9, 11, 29, 47, 57]. However, we also found that low threat labels frequently evoked affective and cognitive responses, and the use of these labels might be important for low income smokers who would be unmotivated by (or avoid) high threat labels. Individuals who perceive a high level of risk but lack self-efficacy might view their susceptibility to diseases as inevitable and take no preventive action [63, 64]. Indeed, we found evidence of fatalistic attitudes among some participants, which might have influenced their reactions to the labels. They might avoid labels portraying a high threat if they lack self-efficacy. Moreover, research has shown that, when confronted with distressing pictures, individuals pay less attention to the persuasive text accompanying the picture [65]. Indeed, when asked why labels with a high threat picture and efficacy text made them feel self-efficacious, our participants focused much of their initial discussion on the threatening picture, rather than the text. To reach a wide range of smokers, labels portraying a range of threat levels might be useful, particularly if text accompanies pictures. Smokers might focus more on the text after acclimation to a distressing picture through repeated exposure over time, but further research is needed to test this hypothesis.

While our study highlights important findings that can assist the development of theory-based graphic warning labels, transferability of the findings to rural or high SES smokers might be limited. Moreover, we are unable to determine if perceptions of threat and efficacy messages on labels would be similar among high SES smokers. These findings should be explored further in qualitative studies and tested in experimental studies with low and high SES smokers to determine the effects and perceptions of efficacy-based labels by SES. Although the small sample size (*N* = 25) might appear to limit the findings, we reached saturation in the responses we received, which is a common criterion for sample size determination in qualitative research. We qualitatively identified and explored several threat and efficacy messages that might influence low income smokers’ risk perceptions and efficacy beliefs, which can be tested in an experimental design. In addition, the cross-sectional design precludes evaluation of the labels’ impact on cessation-related attitudes and behaviors. Participants’ reporting of their perceptions of the labels and labels’ influence on their beliefs has value in elucidating the thought processing and decision-making that occurs when faced with a novel health warning message, but our study cannot make definitive conclusions on the translation into behavior change. Lastly, the labels were explored in a research facility, and so further study of the labels within real-world settings, in which smokers’ interactions with the packs can be observed or measured, would be beneficial.

Despite these limitations, the use of well-established theories to develop and explore warning labels might contribute to the theoretical generalizability of the findings and to methods for developing future labels. The theory used in this study (EPPM) provided a useful lens to investigate the influence of labels on individuals’ risk perceptions and self-efficacy beliefs. However, it would be useful for future work among low SES populations to expand to other individual and environmental characteristics that might affect the influence of labels in this population, such as social norms and perceptions of risk from smoking relative to other risks in their environment like drug use, violence, and food insecurity. Indeed, current warning labels emphasize addiction as the main reason for smoking without consideration of the social and cultural context [66]. Consideration of this greater context is particularly important for low SES populations who face greater barriers to cessation [41, 42].

## Conclusions

Erosion of label effectiveness over time means that new labels need to be developed and implemented periodically [4]. Our novel findings suggest new ways to design labels with efficacy and threat messages to enhance the acceptance and impact of labels. In particular, narratives that allow smokers to vicariously experience characters’ quit successes might be effective. Self-efficacy and response efficacy messages on labels can play a role in efficacy beliefs, either by influencing them or making previously held beliefs salient. Moreover, our findings could aid in the development of graphic warning labels to address smoking disparities among low SES populations.
